# A Quantitative Comparison of Single-Dye Tracking Analysis Tools Using Monte Carlo Simulations

**DOI:** 10.1371/journal.pone.0064287

**Published:** 2013-05-30

**Authors:** Laura Weimann, Kristina A. Ganzinger, James McColl, Kate L. Irvine, Simon J. Davis, Nicholas J. Gay, Clare E. Bryant, David Klenerman

**Affiliations:** 1 Department of Chemistry, University of Cambridge, Cambridge, United Kingdom; 2 Department of Veterinary Medicine, University of Cambridge, Cambridge, United Kingdom; 3 MRC Human Immunology Unit, Nuffield Department of Clinical Medicine, John Radcliffe Hospital, University of Oxford, Oxford, United Kingdom; 4 Department of Biochemistry, University of Cambridge, Cambridge, United Kingdom; Hungarian Academy of Sciences, Hungary

## Abstract

Single-particle tracking (SPT) is widely used to study processes from membrane receptor organization to the dynamics of RNAs in living cells. While single-dye labeling strategies have the benefit of being minimally invasive, this comes at the expense of data quality; typically a data set of short trajectories is obtained and analyzed by means of the mean square displacements (MSD) or the distribution of the particles’ displacements in a set time interval (jump distance, JD). To evaluate the applicability of both approaches, a quantitative comparison of both methods under typically encountered experimental conditions is necessary. Here we use Monte Carlo simulations to systematically compare the accuracy of diffusion coefficients (D-values) obtained for three cases: one population of diffusing species, two populations with different D-values, and a population switching between two D-values. For the first case we find that the MSD gives more or equally accurate results than the JD analysis (relative errors of D-values <6%). If two diffusing species are present or a particle undergoes a motion change, the JD analysis successfully distinguishes both species (relative error <5%). Finally we apply the JD analysis to investigate the motion of endogenous LPS receptors in live macrophages before and after treatment with methyl-β-cyclodextrin and latrunculin B.

## Introduction

Over the past decade the development of more photostable fluorophores [1]–[3] and increasingly sensitive cameras has led to a rise in studies of the motion and spatial organization of cell surface receptors using single-particle tracking (SPT) [4]–[6]. Observing individual particles can be very informative by capturing rare events and returning the distribution of a given variable rather than the ensemble average. All SPT experiments require data collection, particle detection and linking of their positions in subsequent frames. Connecting corresponding particle images in successive frames is no trivial task, and consequently numerous studies have focused on developing tracking algorithms which can deal with situations such as fluorophore blinking, focal drift, and the merging or splitting of trajectories [7]–[9].

Once trajectories are identified, the number of mobile populations present in the experimental data and the distribution of a suitable quantity describing the motion, such as the diffusion coefficient needs to be determined. Although the theory underlying Brownian motion and thus the diffusion of membrane proteins is well-established mathematically [10], in practice the interpretation and extraction of biological information is often challenging [11], [12]. In particular, trajectory lengths can be limited due to photobleaching when minimally-invasive labels such as dye-conjugated Fab fragments of an antibody or ligands are used. While there are many analysis strategies for long trajectories, there is currently a need for a robust analysis of short trajectories obtained from these experiments.

**Figure 1:**
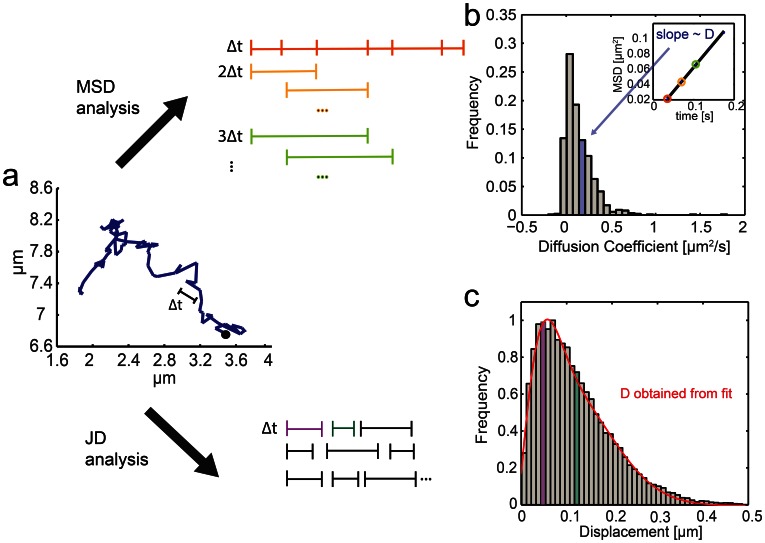
Principle of the mean-square displacement (MSD) and jump distance (JD) analyses. *(a)* A typical single trajectory of receptor-bound LPS recorded in live macrophages (Δ*t* = 33 ms). Trajectory data can be analyzed either by MSD or JD analysis. For MSD analysis, an average of the mean-square displacement is calculated over all points of the individual trajectory for multiples of the smallest resolved time intervals (Δ*t*, *2*Δ*t, 3*Δ*t* etc). The MSD plot over *n*Δ*t* is linear and the gradient is directly proportional to the diffusion coefficient. *(b)* Diffusion coefficients are obtained for all single trajectories and typically presented in histograms. For a random walk with a single diffusion coefficient and long trajectories this distribution is centered around the diffusion coefficient. Multiple mobility populations can be resolved in principle, however a reliable dissection requires a fairly large data set (Note S3 in File S1). The JD analysis plots a histogram of all particle displacements within a fixed time interval Δ*t* for all trajectories. *(c)* Fitting Eq. 7 to the distribution of the displacements yields the minimum number of diffusion coefficients needed to describe the motion of the particles in the system.

Diffusion coefficients are typically obtained by plotting the mean-square displacement (MSD) for a given time lag Δ*t* as a function of Δ*t* [13]–[16]. In the case of simple Brownian motion the gradient of the curve is proportional to the diffusion coefficient *D*. To reduce statistical scatter the MSD is best calculated as the average over all points separated by Δ*t* within a trajectory (Fig. 1, *b*) [10]. The advantage of the MSD analysis it that individual diffusion coefficients are obtained for each trajectory of a given ensemble. However, averaging over an entire trajectory can obscure transitions between different types of motion [11]. An MSD analysis is particularly inaccurate if the length of individual trajectories is short because the time lag becomes a substantial fraction of the trajectory. Saxton showed that if a short range diffusion coefficient is calculated from short trajectories (<32 steps) the distribution of *D* is so wide that measurements of *D* can become useless [17], [18].

For systems for which short trajectories and motion changes are expected an alternative way to analyze the particles’ motion has been employed, referred to as jump-distance (JD) analysis [19]–[25]. Here, the probability of a particle traveling a specific distance within a set time interval is evaluated and fitted by a theoretically-derived probability distribution (Fig. 1, *c*). The main benefit of this approach is that subpopulations of particles with a different probability distribution for their jumps can be easily accounted for by extending the analytical expression used to fit the distribution to multiple populations with distinct values of *D* and fractions. The drawback of this analysis is the loss of single-trajectory information; instead, a single *D* for an ensemble of trajectories with the same mobility is obtained.

In this work we have compared both commonly-used methodologies to test their performance under realistic experimental conditions. We first used simulations to test for which particle densities a local nearest-neighbor based tracking algorithm gives satisfactory results. We then went on to compare MSD and JD analysis, and found that for one diffusing population both return accurate D-values if the mean displacement is large compared to the localization precision (relative errors between 3% and 5%). In contrast to the MSD analysis, the JD analysis also yields accurate diffusion coefficients with small relative errors (6% and 5% respectively) for heterogeneous population of diffusing species and trajectory-inherent motion changes. Finally, we apply the JD analysis to the trajectories of endogenous lipopolysaccharide (LPS) receptors in the plasma membrane of mouse macrophages. Previous studies have found these receptors to be partially immobile. Our results demonstrate that the JD analysis is able to dissect two diffusing populations. Furthermore, this analysis enables us to detect that the motion of the mobile fraction is slowed down by Methyl-β-cyclodextrin (MβCD) and is unaffected by the disruption of the actin cytoskeleton.

## Materials and Methods

### Monte Carlo Simulations

Code written in MATLAB (R 2011b, The MathWorks, Natick, MA) was used to generate simulated data. First, we created a high resolution matrix containing the initial, noise-free particle start positions to generate well separated spots with a given minimum nearest neighbor distance. Then, particle positions in subsequent frames were simulated according to a full continuum model as described in [26]. Briefly, the step size or jump distance *r* a particle was moved in the subsequent frame was a random variable chosen from the two-dimensional Brownian probability distribution [25], [27]

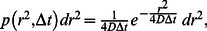
(1)with *D* being the diffusion coefficient and Δ*t* the time interval between frames. The angle of jump θ was another random variable between 0 and 2π, and the particle was moved *dx* = *r* cos(θ) in *x*- and *dy* = *r* sin(θ) in *y*-direction, respectively. The trajectory length *l* was set to 30 image frames similar to the typical *l* obtained from experimental data (Table 1). Since objects viewed through a microscope are distorted by the point spread function of the objective, we convolved the high-resolution matrix with a Gaussian point spread function of a 1.5-pixel radius resembling real data. Next, typical background and Poissonian and Gaussian noise was added to mimic typical EM-CCD signals. For the simulation of two populations, two probability functions with different D-values (*D_m_*(Ground Truth, GT) = 0.1 µm^2^/s, *D_im_*(GT) = 0.02 µm^2^/s) were used. If trajectories with an internal motion change were simulated, a motion change would occur either once or twice within a trajectory, and the new motion would last for at least 5 frames.

**Table 1:** Extracted parameters, diffusion coefficients and fractions for all experimental conditions.

	# cells	# tra-jectories	l* [frames]	a [µm]	D_m_ * [µm^2^/s]	D_im_ * ^† †^[µm^2^/s]	f_m_ * [%]	d ‡ [nm]	a/d	β
Control	40	1465	22±3	1.7±0.4	0.14±0.02	0.015±0.007	73±4	136±10	13±3	5±2
Latrunculin	46	1622	21±3	1.7±0.3	0.14±0.01	0.013±0.003	70±3	136±50	13±3	5±2
MβCD	33	1287	25±3	1.4±0.5	0.07±0.02	0.011±0.003	69±4	96±14	15±5	4±2

*l* denotes the trajectory length,

* Trajectories were randomly grouped in 10 subsets and their JD distributions fitted to equation 7. Errors are given by the standard deviation of the obtained D_m_-, D_im_- and f_m_-values. ^†^Note that the immobile population is static within the localization precision σ = 25±10 nm (Note S4 and Fig. S8 in File S1). SNR = 11 for all cell experiments and 14 for the experiments to determine the localization precision.

‡ *d* = 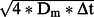, with Δ*t* = 33 ms for all experiments.

### Single Particle Tracking and Trajectory Analysis

Custom-written MATLAB code was used to detect simulated or real particles in each image frame, determine their positions with sub-pixel accuracy, and subsequently link the extracted particle positions using an implementation based on the work by Crocker and Grier [28]. For a detailed description see Note S1 and Fig. S5 in File S1. Obtained trajectories were analyzed using either an MSD or a JD analysis: For the MSD analysis, the mean-square displacement over the first 5 time intervals was calculated and individual diffusion coefficients obtained, using the linear relationship between MSD and Δ*t.* For the JD analysis, the distances between subsequent frames, the so-called jump distances, were analyzed and diffusion coefficients of particle ensembles were obtained by curve fitting [20], [21].

#### Mean-square displacement (MSD) analysis

MSD-values were calculated for each individual track 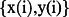 using the method described by Qian et al. [10] and Saxton [17] where *MSD(n*Δ*t)* for a given time lag *n*Δ*t* is defined as the average over all points with that time lag
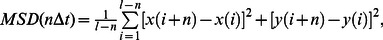
(2)with *l* denoting the trajectory length and Δ*t* the time step between frames. Then short range diffusion coefficients *D* were obtained for each trajectory using a linear weighted fit for *n*≤5 to the equation
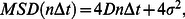
(3)assuming errors are approximately normally distributed, the offset corresponds to 4σ2 with σ being the localization precision [23]. Only n≤5 time lags were considered for the analysis since the maximum time lag should not exceed a quarter of the total trajectory length (approx. 20 steps for our data) [17]. The gradient of the linear fit is proportional to D. A histogram of individual D-values is obtained and the mean value <D> used to characterize the motion of the ensemble.

#### Jump Distance (JD) Analysis

The probability *p(r^2^,*Δ*t)dr^2^* that a particle starting at *r_1_* = 0 will be encountered within a shell of radius *r* and a width *dr* at time Δ*t* is given for two-dimensional Brownian motion by [25], [27]:
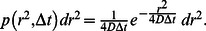
(4)


This probability distribution can be obtained experimentally by counting the jump distances within intervals [*r,r+dr*] traveled by single particles in Δ*t*. The diffusion coefficient of a particle ensemble can then be determined by fitting the above equation to the experimental data. For the purpose of fitting, the integrated distribution
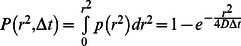
(5)is more convenient, as it is independent of the choice of the bin sizes [29].

If *m* species are present, a sum of *m* terms is used
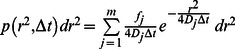
(6)


Here, *f_j_* the respective fraction of particles in mobility mode *j* and *D_j_* the respective diffusion coefficient.

For the integrated distribution, it holds [20]

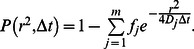
(7)


To evaluate the goodness of the fit, the residual is inspected. To prevent over-fitting we recommend starting with the simplest model (*m* = 1) fitting to the data and subsequently checking the fit residuals for any systematic deviation. If a systematic deviation is observed *m* should be increased and a more complex model fitted to the data, and the simplest model whose fit result shows no systematic deviation should be chosen [25], [30], [31].

### Cell Culture Procedures

The mouse macrophage cell line *RAW 264.7* (ATCC TIB-71, European Tissue Culture Collection) was cultured and maintained in RPMI (Invitrogen) containing 1% penicillin/streptomycin (100 times, Invitrogen), 1% L-Glutamine (200 mM, Invitrogen) and 10% heat-inactivated fetal bovine serum (HyClone, ThermoScientific) in an incubator at 5% CO_2_ and 37°C. Cells were grown to 60–70% confluence before splitting. Mechanical force was used for their detachment, and after every fourth passage the cells were transferred to a fresh culture flask (BioGreiner).

### Sample Preparation for Microscopy

LPS purified from *E. coli* and labeled with AlexaFluor 488 (Invitrogen) was stored at −20°C at a stock concentration of 1 mg/ml. The labeled LPS was tested with respect to TLR4 activation in HEK cells transfected with human TLR4, MD-2 and CD14, and found to activate cells to a similar extent to unlabeled LPS (Note S5 and Fig. S9 in File S1). RAW 264.7 cells were seeded on 48-well plates (UpCell plates, NUNC) at a density of 6×10^5^ cells per well in culture medium and stimulated 24 hours later. Prior to cell stimulation, LPS aliquots were sonicated in two 30-second bursts separated by a 1-minute pause. Cells were then incubated on the plate in medium containing LPS at 1.8 µg/ml for 15 minutes on ice. For some experiments the cells were pretreated with 10 mM Methyl-β-cyclodextrin in RPMI supplemented with 0.5% FCS and 1% L-Glutamine for 2 hours, or with 2.5 µM latrunculin B (Biomol International, Exeter, UK) in serum free medium for 20 minutes.

Following incubation with LPS, cells were then taken off the plate by aspiration, and washed 3 times with ice cold RPMI and the cells recovered by centrifugation (600 g, 2 min, 4°C). The cell pellet was resuspended in RPMI supplemented with 1% FCS, and dropped onto a cover slip housed in a home-made chamber (total volume of medium: 1 mL RPMI/1% FCS), pre-warmed to 37°C for imaging. The microscope coverslips had been cleaned for 10 minutes with Argon plasma (PDC-002, Harrick Plasma, Ithaca N.Y.). A stage incubator was used to maintain temperature and CO_2_ content (37°C, 5% CO_2_) throughout the entire measurements and all data were taken within the first 20 minutes.

### TIRFM Experimental Setup

Imaging was performed using total internal reflection fluorescence microscopy (TIRFM). A diode laser operating at 488 nm (PC13589, Spectra Physics) was directed into a TIRF objective (60× Plan Apo TIRF, NA 1.45, Nikon) mounted on a Nikon Eclipse TE2000-U microscope off the optical axis so that it impinged on the sample above the critical angle. Fluorescence collected by the same objective was separated from the returning TIR beam by a dichroic (FF500/646-Di1, Semrock), and filtered using Dual-View™ (Photometrics) mounted filters. The images were recorded on an EMCCD camera (Cascade II: 512, Photometrics) operating at −70°C. Data were acquired at the rate of 28.6 frames s^−1^ using Micromanager [32], and the pixel size of the camera was 106 nm. For each treatment, we collected approx. 1300 trajectories in three independent experiments which lasted an average length of 23 frames.

## Results and Discussion

### Testing of Nearest-neighbor Tracking Function

One key step in SPT - connecting particle images in a sequence of frames - is complicated by high particle density. If the particle density is sufficiently low, frame-to-frame particle correspondence is almost unambiguous. At these low densities, tracking functions based on a local nearest-neighbor approach have been used extensively [15], [28], [33], [34], yet to date there has been no systematic test of the performance of such an algorithm as a function of particle density under realistic conditions. The density of a sample can be characterized by the average nearest neighbor distance *a* between particles. Linking particles into trajectories over time is feasible if the average particle displacement *d* within the time interval Δ*t* is sufficiently smaller than *a* (*d*<<*a)*. In that case, a tracking function based on a nearest-neighbor approach is expected to perform well. For *d* ≈ *a,* particle assignment becomes ambiguous because the distance between some nearest-neighbors is smaller than their average displacement between frames. The ratio *a/d* of a data set can thus be used to test whether a nearest neighbor approach is applicable.

**Figure 2:**
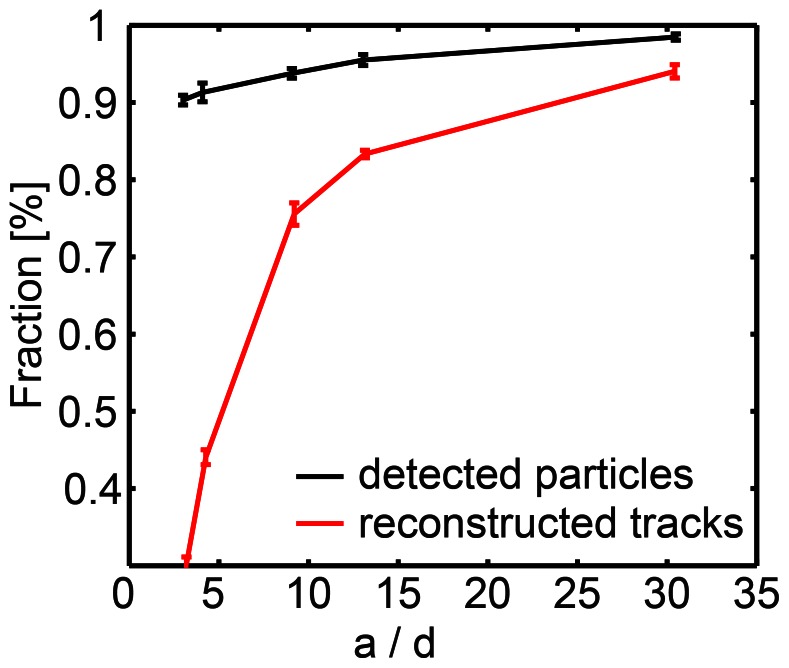
Validation of tracking function on simulated data. The fraction of detected particles and fully reconstructed tracks (number of true positives/ground truth (GT)) as a function of the ratio of the average nearest neighbor distance over the average displacement *a/d*. Image stacks with 150 particles were simulated over 30 frames. In the first image frame particles were located at a distance 5×radius of Airy disk from each other and then moved assuming Brownian motion in subsequent frames. *d* was varied. Each data point shows the mean value of 50 repetitions.

Therefore, in order to test the performance of the nearest-neighbor approach implemented by Crocker and Grier [28] with varying *a*/*d*, we simulated data with a signal/noise ratio (SNR) and a trajectory length *l* matching experimental data (*l* = 30 steps, SNR = 6). In the first image frame particles were located at a defined distance and moved a distance *d* according to equation (1). Since the detection efficiency for isolated particles is 100% for SNR-values >3 (Note S2 and Fig. S6 in File S1), the fraction of detected particles only slightly depends on *a*/*d* (Fig. 2, *black curve*). The slight drop towards low *a/d* values (large *d*) is due to the probability that two particles get close together if *d* is large. Then their point spread functions overlap and both particles cannot be resolved. The fraction of fully reconstructed trajectories (Fig. 2, *blue curve*), however, is affected more strongly; the reconstruction requires every single particle position to be detected and linked correctly. For *a*/*d* = 10, we find that a nearest neighbor approach fully reconstructs 80% of the tracks and conclude that it is suited to analyze data samples with *a*/*d* ≥10. For *a*/*d* = 5 approximately 50% of the tracks are recovered; this is well in line with a previous study by Jaqaman *et al.* [35] which suggests that local nearest neighbor approaches fail for *a*/*d* <5. For *a*/*d* <10, computationally more expensive algorithms should be used instead [7], [9].

### Computer Simulations Determine the Accuracy of JD and MSD Analysis Under Real Experimental Conditions

**Figure 3:**
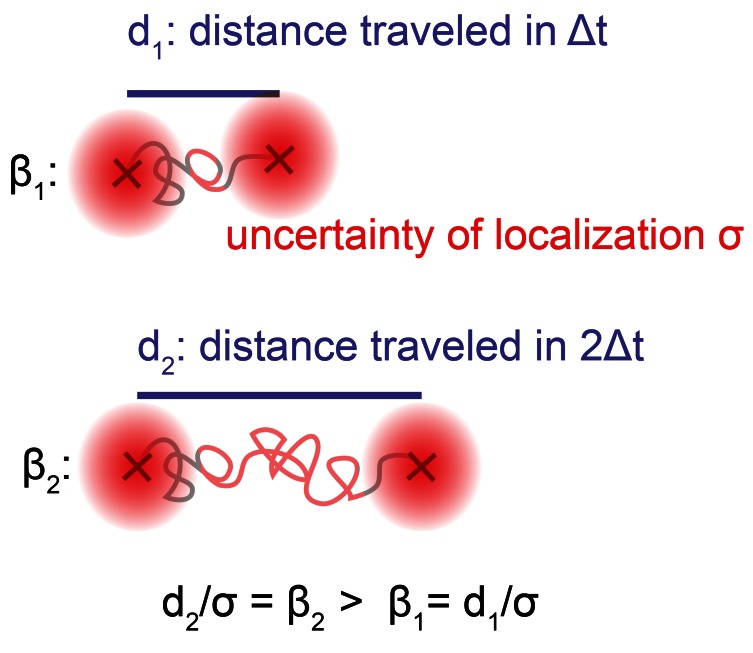
Illustration of the parameter *β*. Depending on the SNR, a particle can be localized within a certain localization precision *σ* (*red circle*). After a certain time interval the particle has traveled a distance *d*. If *σ* is a substantial fraction of the distance *d* (i.e. *β* is small), the measurement of *d* is imprecise, leading to errors in the determination of the diffusion coefficient. Increasing the time interval Δ*t* and thus *β* allows a more precise measurement of *d*.

**Figure 4:**
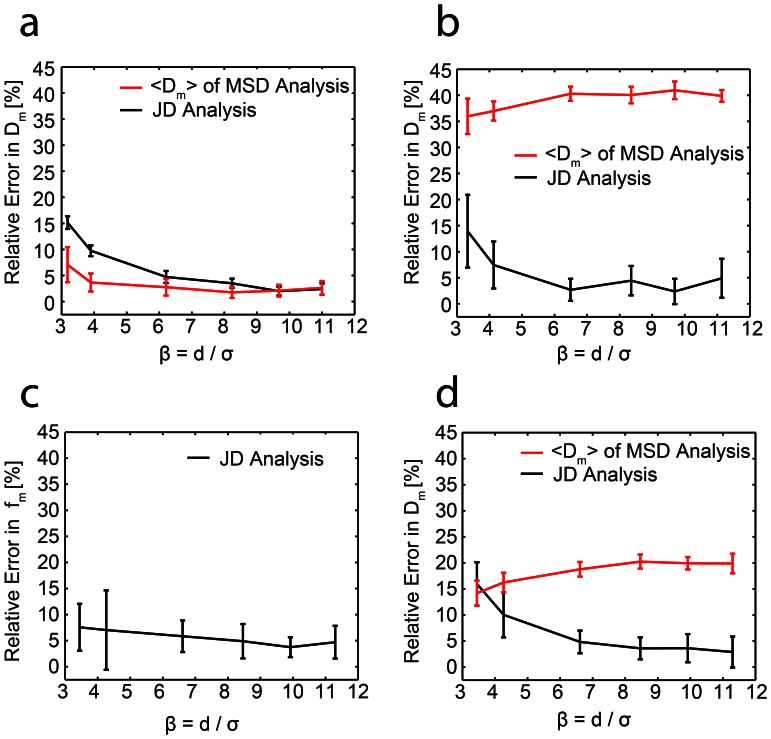
Comparison of JD analysis and MSD analysis on simulated data. Simulations with varying mobile and immobile populations were used to compare the performance of the JD analysis *(black curves)* to the standard MSD approach *(red curves). (a,b,d)* The results shown are the relative error |*D_m,_ (output)−D_m,_(ground truth, GT)|/D_m_ (GT)* in the diffusion coefficient of the mobile population as a function of the parameter *β* and *(c)* the relative error *|f_m_ (output)−f_m_(GT)|/f_m_ (GT)* in the mobile fraction as a function of the parameter *β*. All results are the mean values of 10 repetitions, with each repetition based on the analysis of 750 simulated trajectories. Error bars represent ±1 standard deviation *(a)* All particles belong to the mobile population. *(b,c)* 50% of the particles are mobile, and 50% immobile *(d)* 50% of particles undergo a motion change (mobile → immobile).

In order to compare the JD to the MSD analysis, we tested the performance of both methods depending on *β,* with *β* being defined as the ratio of two experimentally accessible parameters: the average particle displacement *d* and the particle's localization precision *σ* (*β = d/σ*). The measurement of the distance *d* a particle has traveled within a given time interval Δ*t (*Δ*t* = Δ*t_exposure_*+Δ*t_delay_)* becomes inaccurate if *σ* is a substantial fraction of this distance (Fig. 3). It is expected that the JD approach is sensitive to *β*, because for the JD approach the localization precision directly affects the jump distance measurement. Furthermore, the JD approach is not sensitive to the trajectory length, because its performance depends on­ the overall number of correctly linked particles. The MSD analysis, on the other hand, is less dependent on *β* and the trajectory length is expected to be a critical parameter, since the MSD(Δ*t*) is obtained as an average of all MSD(Δ*t*) over the entire trajectory [10]. As short trajectories are typically obtained in single-dye tracking experiments, we were interested to investigate in which regime of *β* the JD analysis provides equivalent results to the MSD analysis. To this end, image stacks with particles undergoing Brownian motion were simulated with an SNR, a particle density and a short trajectory length close to experimentally obtained values (Movies S1 and S2). We started with the most trivial case in which only one mobility population with a diffusion coefficient *D_m_(ground truth, GT)* = 0.1 µm^2^/s was simulated. We varied Δ*t_delay_* to cover values of *β* ranging from 3 to 12. Trajectories were obtained from the simulated data using the described spot detection and tracking function, and the diffusion coefficient *D_m_(output)* extracted using either the JD or MSD approach. Then, the relative error 
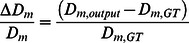
of the output diffusion coefficient was compared for both methods. Fig. 4 *a* shows that for the case of one mobile population, the JD analysis reconstructs the diffusion coefficient as accurately as the MSD analysis if *β* exceeds a value of 6 (relative errors Δ*D_m_/D_m_* between 3% and 5%, Fig. S7 *b,c* and Note S3 in File S1 for representative data and fits).

### Different Mobility Modes and Motion Changes can be Reliably Dissected by JD Analysis

In the case of one mobility population, the JD is as accurate as the MSD analysis if *β*>6. However, real receptor motion is expected to be less homogenous due to the organization of the plasma membrane: in a mosaic-like membrane receptors are expected to move in a heterogeneous fashion, sub-populations with varying diffusion coefficients may exist and transitions between different types of motion may occur on the time scales of the particle tracking [36], [37]. To test the performance of both trajectory analyses under more realistic conditions, two mobility populations undergoing Brownian motion were simulated as above. 50% of the particles belonged to a mobile species (*D_m_(GT)* = 0.1 µm^2^/s) and 50% to an immobile species (*D_im_(GT)* = 0.02 µm^2^/s). For *β*>6 the JD analysis yields a relative error in *D_m_* of approximately 6% whereas the MSD analysis fails to reconstruct *D_m_* reliably (Δ*D_m_/D_m_* ≈ 40% for all *β,* Fig. 4 *b*). Additionally, the JD analysis reliably extracts the fractions *f_m_(GT)* = 0.5 of the mobile population, with relative errors of *f_m_* between 4% and 8% (Fig. 4 *c*). We also tested how a change in the mobile fraction *f_m_* (constant *β*) affected both the JD and MSD analysis (Fig. S1 in File S1). Not surprisingly, the relative error in *D_m_* for the MSD analysis increased drastically for an increasingly smaller mobile fraction *f_m_* whereas the JD analysis still returns reliable values for *D_m_*. We would like to add that theoretically two populations are resolvable by the MSD approach since the histograms of the diffusion coefficients (Fig. 1 *b*) should show two peaks representing two different D-values. However, under the conditions examined mimicking typical diffusion data properties, the obtained histograms show that the distributions of D-values are too wide (due to short trajectories) to resolve the peaks for a typical data set of 750 trajectories (Fig. S7 *d* and Note S3 in File S1). The JD analysis, however, already performs well for a data set of this size.

Next, simulations were used to compare the performance of both approaches with half of the particles undergoing motion changes (mobile → immobile, or mobile → immobile → mobile) within a trajectory (Fig. S4 in File S1). The dependence of Δ*D_m_/D_m_* on *β* shows the same tendency as for the case of two discrete mobile and immobile populations discussed above**.** For *β*>5, the JD approach recovered *D_m_* with a relative error of approx. 5%, whereas the MSD approach gave a higher relative error (Δ*D_m_/D_m_*>18%, Fig. 4 *d*). Changing the fraction of the particles undergoing a motion change (constant *β*) affected both the JD and MSD analysis. We found that if more than 30% of the particles undergo a motion change, the JD analysis outperforms the MSD analysis (Fig. S2 in File S1).

### Performance for Experimental Data: Native CD14 and TLR4 Organization

Investigating the motion of endogenous cell surface receptors is challenging but also rewarding as the receptors can be studied in a minimally-perturbed and thus more relevant environment. Two receptors of particular interest are CD14 and Toll-like Receptor 4 (TLR4). Both play a fundamental role in generating an immune response against infection by recognizing LPS, a component of the Gram-negative bacterial cell wall [38]. Over-activation of TLR4 by Gram-negative bacteria can lead to sepsis, making antagonism of this receptor an important therapeutic goal [39]. TLR4 dimerization is known to be required for activation of signaling pathways; however how LPS-bound CD14 and TLR4 behave within the cell membrane is unclear.

Therefore we decided to visualize endogenous CD14 and TLR4 receptor motion by using fluorescently labeled LPS (AlexaFluor 488, see Movies S3 and S4 for representative data). Since LPS did not bind to CD14−/− macrophages (Fig. S3 in File S1), we assume that the detected LPS molecules are either bound to CD14 or TLR4 after transfer via CD14. First we characterized all data sets to be confident that our tracking and JD analysis algorithms would be applicable (measuring the SNR, localization precision, average nearest neighbor distance and *β*, Table 1). Furthermore, visual inspection of the data revealed motion changes (a representative trajectory is shown in Fig. S4 in File S1), indicating that an MSD analysis would not be appropriate. This is in agreement with a previous study [40] of TLR4 receptor diffusion using FRAP which have also found that a fraction of TLR4 receptors is immobile.

**Figure 5:**
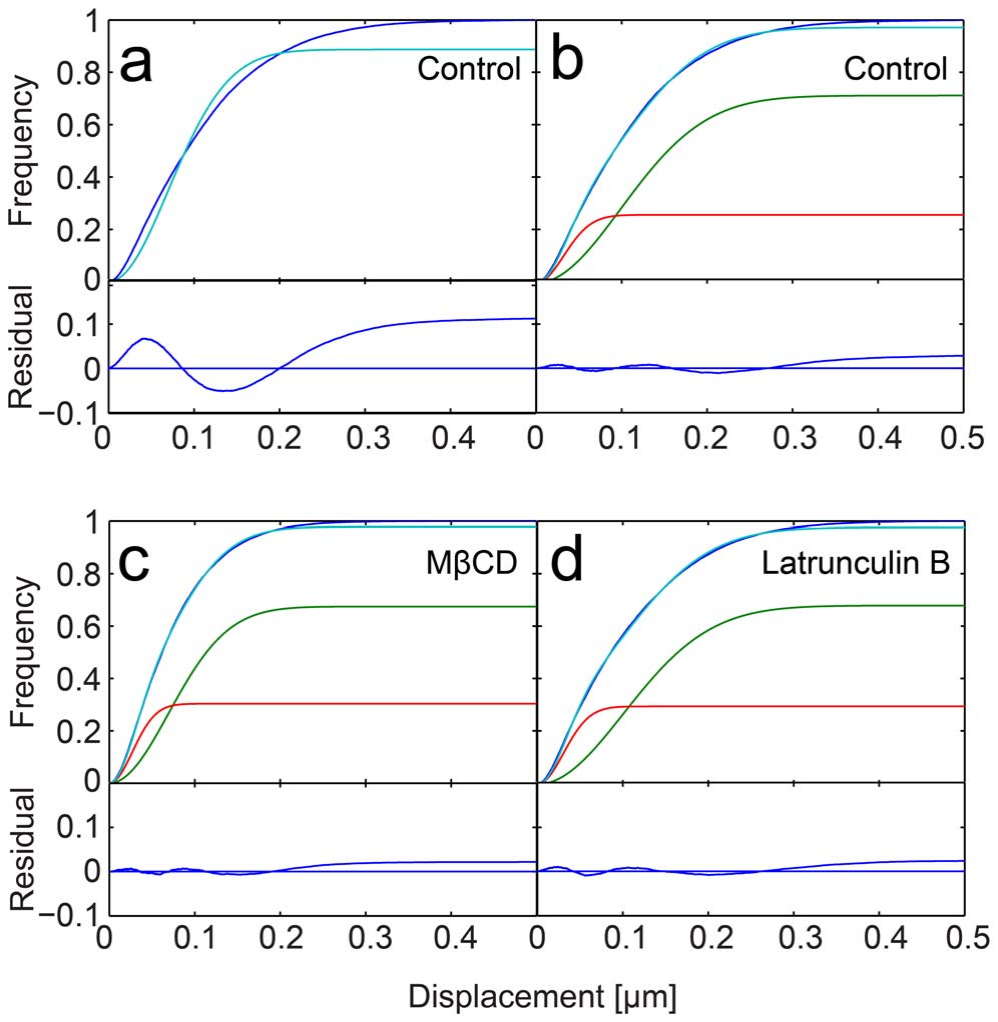
Cumulative histograms of jump distances of LPS-bound receptors in the plasma membrane *(blue line).* Best fits according to Eq. 7 are shown *(cyan)*. *(a)* The cyan line represents the fit result assuming one species is present. The residual shows a clear deviation from the data. *(b)* The same data fitted assuming two species are present (components of the fit are shown in *green* for the mobile and *red* for the immobile population). The residual shows no systematic deviation of the fit from the data. Note that the deviation of the fit from the histogram towards larger displacements is also found in our simulated data (Fig. S7 and Note S3 in File S1) and thus does not indicate a deviation of the data from Brownian motion. *(c)* MβCD treated and *(d)* latrunculin B treated macrophages fitted assuming two mobility populations. The values for the diffusion coefficients and fractions can be found in Table 1.

The JD distribution revealed that there are at least two populations with different mobility modes present (Fig. 5 a,*b*, Table 1), as fitting the data assuming one population fails. These two populations are unlikely to correspond directly to the two receptors since CD14 receptors are much more abundant than TLR4 (40-fold excess of CD14 [41]). The faster-moving particles (two-thirds of the receptors) moved with a diffusion coefficient of *D_m_* = (0.14±0.02) µm^2^/s, all other particles were immobile within our localization precision (Note S4, Fig. S8 in File S1).

In previous work it was found that treatment with the compound MβCD used to extract the cholesterol from the plasma membrane slows down the motion of glycophosphatidylinositol-(GPI-)anchored receptors by a factor of two [42]. We thus probed whether MβCD had a similar effect on LPS receptor motion since CD14 is also a GPI-anchored protein [43]. For macrophages pretreated with MβCD two populations with different mobility modes were recovered, and the immobile fraction measured for cholesterol depleted cells was similar to the control sample, see Fig. 5 *c* and Table 1. The diffusion coefficient of the faster population dropped by 50% for treated cells to a value of *D_m_* = (0.07±0.02) µm^2^/s which is in excellent agreement with the study by Vrljic *et al*. [42]. However, this result is seemingly in disagreement with the expected change in membrane fluidity upon treatment with MβCD, as the membrane should be more fluid in the absence of cholesterol and thus the receptor motion should be faster. Therefore we assume that the observed reduction in receptor mobility after MβCD treatment is due to a previously reported side effect of the drug: MβCD was found to strengthen the adhesion of the cytoskeleton to the plasma membrane [44] which explains the reduced receptor mobility. Thus, the effect of MβCD on cytoskeletal organization dominates the effect of cholesterol depletion, and hence it should not be used to investigate whether the lipid composition is of importance for receptor organization and signaling. Instead, membrane cholesterol levels could be manipulated by metabolic inhibition using compounds such as compactin [45].

Finally we also investigated the effect of the underlying cytoskeleton on receptor motion by treating the cells with latrunculin B, a drug which is commonly used to depolymerise the actin cytoskeleton. Interestingly, receptor diffusion was found to be unaffected by this treatment (*D_m_* = 0.14±0.01 µm^2^/s, Fig. 5 *d* and Table 1). As CD14 is a GPI-anchored protein, this fits previous observations that some actin disruption agents such as Cytochalasin D do not influence the motion of GPI-anchored proteins [42].

### Conclusion

In this study we have investigated how information on particle diffusion can be reliably extracted from short single-dye trajectories. Using Monte Carlo simulations, we have shown that a nearest-neighbor approach leads to a successful reconstruction of the trajectories if the average nearest neighbor distance *a* is ≥10 times the average displacement *d*. If *a/d* <10, a different tracking approach should be chosen [46], and its suitability tested by simulations or the plateau region criterion suggested by Wieser *et al* [47]. Ensuring reliable trajectory formation is particularly important if the density of the molecule of interest cannot be varied experimentally, for instance if the diffusional behavior of endogenous receptors is investigated.

If short trajectories of one diffusing population are analyzed, and if the localization precision is not a substantial fraction of the particles’ mean displacement (*β*>6), we have demonstrated that both MSD and JD analysis yield accurate D-values (Δ*D/D* = 3–5%). For two populations with different D-values, or a population switching between two D-values, the MSD analysis fails to reconstruct the D-values reliably whereas the JD analysis yields accurate D-values (Δ*D*/*D* ≈ 6% for *β*>6). In both latter cases, a complete reconstruction of trajectories is crucial for the accuracy of a MSD analysis whereas the accuracy of a JD analysis is less affected by failures in particle linking (falsely linked particles contribute one false step size to the histogram as opposed to a false D-value of an entire trajectory). A JD analysis can therefore be advantageous in an experimental situation for which false particle links cannot be completely avoided due to high receptor densities. We suggest that the JD analysis should be used for an initial analysis of experimental data if the underlying number of diffusion populations is unknown. Even if receptor diffusion can be described using a single D-value, the labeling method, e.g. using fluorophore-tagged antibodies or ligands, can introduce artificial motion changes if the off-rate of the antibody or ligand cannot be neglected during the experiment.

We have chosen a sample size and mean trajectory length typically obtained for single dye tracking studies. In general, a JD analysis improves with the number of correctly linked particles which could be achieved either by longer trajectories or a larger sample size. For a single population, JD and MSD approach are expected to yield similar results for *β*>6 even if the sample size and trajectory length is increased. For trajectory length longer than 100 frames (which is hard to achieve for single-dye tracking), we would recommend a MSD approach as it has the potential to extract additional information [11]. For two populations a significant increase of the sample size is required to distinguish motion modes reliably with an MSD analysis for *β*>6. An approx. 5-fold larger sample size will lead to two distinct peaks in the distribution of diffusion coefficients (see Figure S7d). In the case of motion changes within a particle trajectory, a JD approach is beneficial for *β*>6, independent of trajectory length and sample size, as MSD analysis obscures motion changes.

Finally we applied the JD analysis to analyze the motion of single-dye labeled endogenous LPS receptors and found that these receptors are partially immobile. While the motion of the mobile fraction is slowed down by MβCD, it is unaffected by the disruption of the actin cytoskeleton.

It is our hope that the principles established in this work will help to guide other researchers in their choice of analysis method for single-dye tracking data and thereby ultimately contribute to a more detailed understanding of membrane protein organization.

The MATLAB code for simulations and analysis software is available upon request, contact L.W. (laura.weimann@gmail.com) or D.K. (dk10012@cam.ac.uk).

## Supporting Information

File S1 Figure S1, Probing the capability of the JD analysis to output accurate diffusion coefficients *D_m_(f_m_)* and to resolve varying mobile fractions *f_m_*. Figure S2, Probing the capability of the JD analysis to output accurate diffusion coefficients *D_m_* varying the fractions of particles undergoing a motion change. Figure S3, LPS does not bind to CD14−/− macrophages. Figure S4, Representative trajectory of a receptor bound LPS molecule undergoing a motion change. Note S1, Spot Detection and Tracking Algorithm (Figure S5). Note S2, Testing of spot detection and localization procedures (Figure S6). Note S3, Comparison of MSD and JD Analysis using simulated data (Figure S7). Note S4, Measuring the localization precision (Figure S8). Note S5, Test of biological activity of AlexaFluor®488 labeled LPS (Figure S9).
(DOCX)

Movie S1 Representative Movie showing simulated receptor motion (raw data).
(AVI)

Movie S2 Same video as Movie S1 after application of a band-pass filter.
(AVI)

Movie S3 Movie typically obtained for AlexaFluor®488 labelled LPS on wild-type macrophages (raw data).
(AVI)

Movie S4 Same video as Movie S3 after application of a band-pass filter.
(AVI)
