# Supplementary material for: A Quantitative Comparison of Single-Dye Tracking Analysis Tools Using Monte Carlo Simulations
Source: PLoS One. 2013 May 30;8(5):e64287. doi: 10.1371/journal.pone.0064287 (PMC3667770; doi:10.1371/journal.pone.0064287)
Supplement: File S1 — Figure S1, Probing the capability of the JD analysis to output accurate diffusion coefficients Dm(fm) and to resolve varying mobile fractions fm. Figure S2, Probing the capability of the JD analysis to output accurate diffusion coefficients Dm varying the fractions of particles undergoing a motion change. Figure S3, LPS does not bind to CD14−/− macrophages. Figure S4, Representative trajectory of a receptor bound LPS molecule undergoing a motion change. Note S1, Spot Detection and Tracking Algorithm (Figure S5). Note S2, Testing of spot detection and localization procedures (Figure S6). Note S3, Comparison of MSD and JD Analysis using simulated data (Figure S7). Note S4, Measuring the localization precision (Figure S8). Note S5, Test of biological activity of AlexaFluor®488 labeled LPS (Figure S9). (DOCX) [file pone.0064287.s001.docx]

A quantitative comparison of single-dye tracking analysis tools using Monte Carlo simulations: Supporting Material

Laura Weimann, Kristina A Ganzinger, James McColl, Kate L Irvine, Simon J. Davis, Nicholas J Gay, Clare E Bryant, David Klenerman.

Contents

**Supplementary Figure 1** Probing the capability of the JD analysis to output accurate diffusion coefficients *D_m_(f_m_)* and to resolve varying mobile fractions *f_m_*

**Supplementary Figure 2** Probing the capability of the JD analysis to output accurate diffusion coefficients *D_m_* varying the fractions of particles undergoing a motion change

**Supplementary Figure 3** LPS does not bind to CD14 -/- macrophages

**Supplementary Figure 4** Representative trajectory of a receptor bound LPS molecule undergoing a motion change

**Supplementary Note S1** Spot Detection and Tracking Algorithm

**Supplementary Note S2** Testing of spot detection and localization procedures

**Supplementary Note S3** Comparison of MSD and JD Analysis using simulated data

**Supplementary Note S4** Measuring the localization precision

**Supplementary Note S5** Test of biological activity of AlexaFluor®488 labeled LPS

**Supplementary Movie 1**

**Supplementary Movie 2**

**Supplementary Movie 3**

**Supplementary Movie 4**

**Supplementary Figures**

**Figure S1**


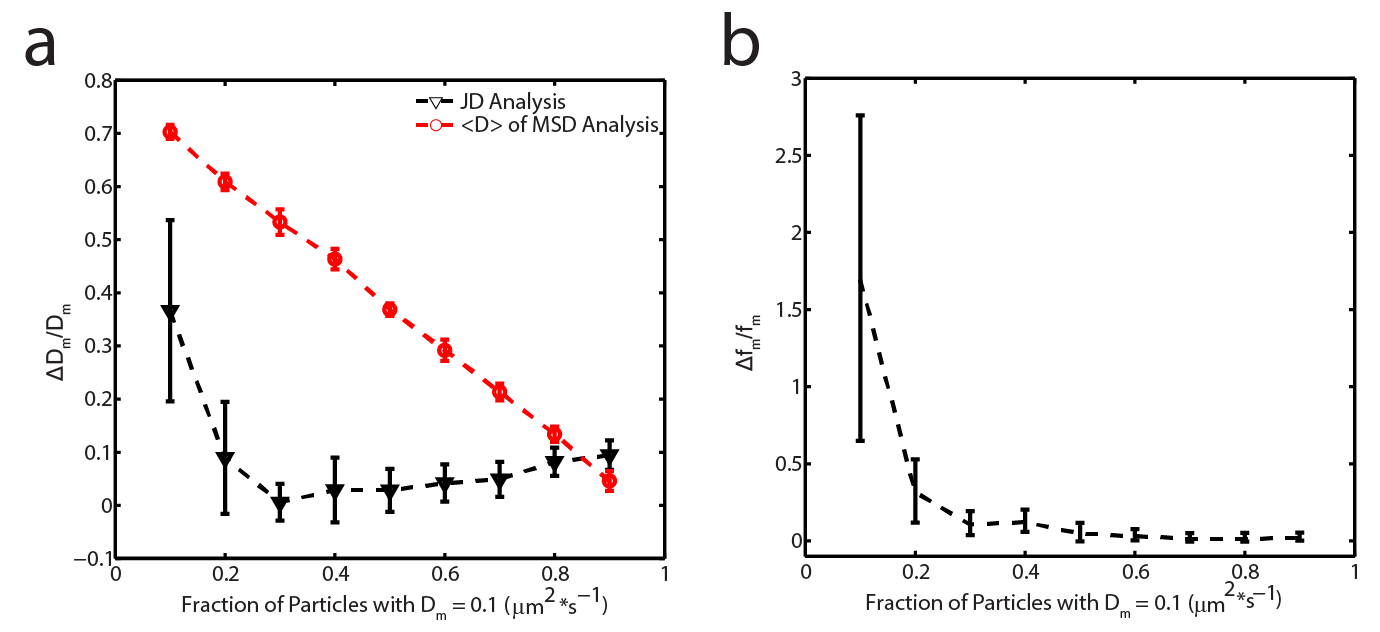


Figure S1 Probing the capability of the JD analysis to output accurate diffusion coefficients *D_m_(f_m_)* and to resolve varying mobile fractions *f_m_* *(a,b)* Simulations with varying fractions *f_m_* of the mobile (*D_m_(GT)* = 0.1 μm^2^/s) and immobile (*D_im_(GT)* = 0.02 μm^2^/s) populations were used to compare the performance of the JD analysis *(black curves)* to the standard MSD approach *(red curves)*. The relative error |*D_m_(output)* - *D_m_(GT)*| / *D_m_(GT)* in the diffusion coefficient of the mobile population is shown as a function of the fraction f_m_. The data was compiled from 50 videos with 150 particles over 30 frames. SNR = 6, *σ* = 29 nm, *a* = 1060 nm, Δ*t* = 33 ms and *β* = 4. Each data point contains 10 videos which equals 750 tracks. The mean is shown and error bars represent one standard deviation. *(a)* As expected, the MSD approach fails to reconstruct *D_m_* reliably if the mobile fraction is small. The JD analysis allows a reliable reconstruction of the diffusion coefficient *D_m_* if the mobile fraction *f_m_* > 20 % with a relative error < 10 %, and also outputs the fraction *f_m_* reliably *(b)*.

**Figure S2**


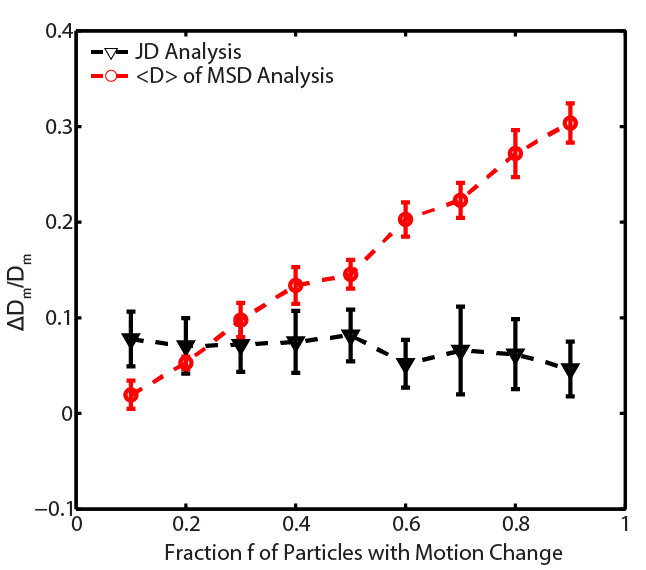


Figure S2 Probing the capability of the JD analysis to output accurate diffusion coefficients *D_m_* varying the fractions of particles undergoing a motion change. Simulations of particles changing their motion (*D_m_(GT)* = 0.1 🡪 *D_im_(GT)*= 0.02 μm^2^/s) and (*D_m_(GT)* = 0.1 🡪 *D_im_(GT)*= 0.02 μm^2^/s 🡪 *D_m_(GT)* = 0.1) were used to compare the performance of the JD analysis *(black curves)* to the standard MSD approach *(red curves)*. The relative error |*D_m_(output)* - *D_m_(GT)*| / *D_m_ (GT)* in the diffusion coefficient of the mobile population is shown as a function of the fraction f. The data was compiled from 50 videos with 150 particles over 30 frames. SNR = 6, *σ* = 29 nm, *a* = 1060 nm, Δ*t* = 33 ms and *β* = 4. Each data point contains 10 videos which equals 750 tracks. The mean is shown and error bars represent one standard deviation. In contrary to the JD analysis, the MSD analysis fails to give correct diffusion coefficients if a fraction larger than 20 % undergoes a motion change.

**Figure S3**


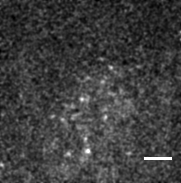

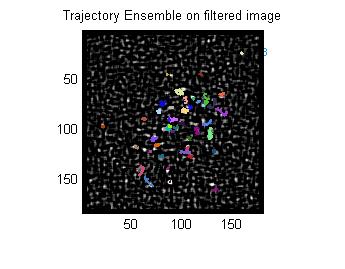

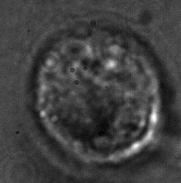


**c**

**bA**

**a**


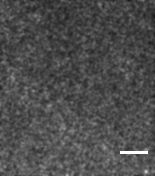

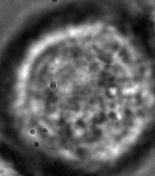


**eA**

**dA**

Figure S3 LPS does not bind to CD14 -/- macrophages. *(a-c)* Alexa®488 labeled LPS binds to wild-type macrophages; *(a)* Single video frame of LPS-labeled surface receptors in wild-type macrophages (Δ*t_exposure_* = 33 ms, raw data). *(b)* Trajectories of receptor (CD14, TLR4) bound LPS shown on band pass filtered image *(c)* phase contrast image; *(d-e)* Alexa®488 labeled LPS does not bind to CD14 -/- BMDM (bone marrow derived macrophages); *(d)* first frame (raw data), *(e)* phase contrast image

**Figure S4**


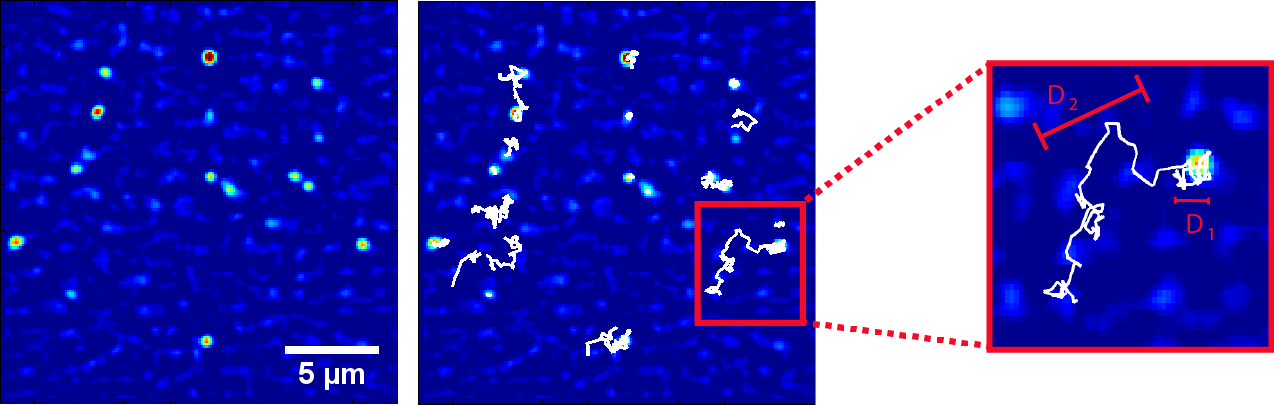


Figure S4 Representative trajectory of a receptor bound LPS molecule undergoing a motion change. *(left)* Single video frame of receptor bound LPS (Δ*t_exposure_* = 33 ms, band pass filtered data). *(middle)* Trajectories plotted on *(left)*. *(right)* Zoom-in on a particle undergoing a motion change. The particle motion changes from being confined to a random walk resembling pattern.

**Supplementary Notes**

**Supplementary Note S1** Spot Detection and Tracking Algorithm

Custom-written MATLAB code was used to detect simulated or real particles in each image frame. The spot detection algorithm implemented was designed to extract sub-pixel locations of diffraction limited points in each image frame. Particles are then linked to trajectories using an algorithm based on the work by Crocker and Grier (1).

Step 1) Initial Image Analysis

We used a band-pass image filter to remove low-frequency modulated background and high-frequency noise typically present in our image data (Fig. S5 *b*). The implemented filtering algorithm [bpass.m] was converted to MATLAB by E. Dufresne and is freely available online (2).

Step 2) Spot Detection and Localization

Particles with intensities above a user-defined threshold were located by calculating their brightness-weighted centroid (Fig. S5 *c*). Optionally the particle’s positions were redefined by fitting two-dimensional Gaussians to the particles. Particles could be discriminated against noise and artifacts on the basis of two criteria (Fig. S5 *d*): 1. spot size - large spots are likely to be artifacts and small spots are likely to be noise - and 2. the local signal-to-noise ratio SNR (exclusion of spots below a user-defined SNR).

The spot detection algorithm is perfectly suited to analyze data with a SNR higher than 3 (Fig. S6). Compared to other spot detection algorithms (3) it is very straightforward to implement and computationally less expensive. In addition, the local noise discrimination makes the algorithm robust against global intensity variations due to uneven TIRF illumination and locally variable background.

As for the band pass filtering, two functions written by E. Dufresne were implemented which are freely available online (Pkfnd.m to find local brightness maxima and cntrd.m to calculate centroid positions, (2))

Step 3) Trajectory Formation

After spot detection trajectories were formed by connecting one particle to its nearest neighbor in the consecutive frame. To this end we implemented an algorithm which calculated the distances between all particles in the nth and (n + 1)^th^ frames. Tracks were then created by linking each particle to its closest partner in the subsequent frame by minimizing the total inter-particle distance. To reduce the complexity of this optimization problem, only distances of particles shorter than a characteristic length scale were calculated.

Furthermore, the possibility of particles disappearing from the observable image plane was taken into account. Missing particles can be added, and if they reappeared sufficiently close to a given trajectory they could be recovered. A more detailed description can be found in (1). A MATLAB implementation by E. Dufresne was used which is available online (2).


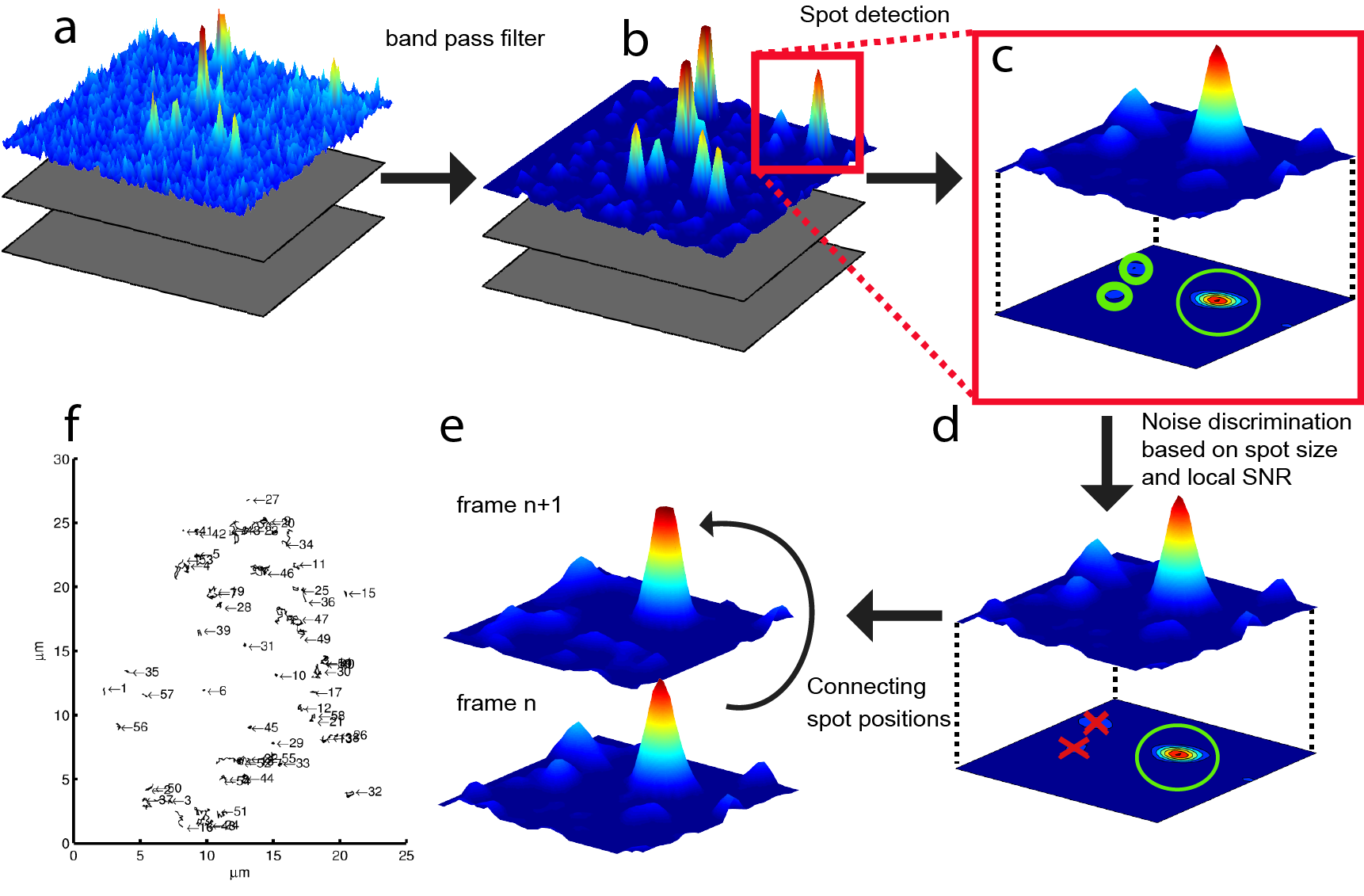
 FIGURE S5 Optimized Spot Detection and Trajectory Formation *(a)* Single video frame of LPS-labeled surface receptors in macrophages (Δ*t_exposure_* = 33 ms, raw data). *(b)* Image from *(a)* after application of a band pass filter (implementation by E. Dufresne (2)). *(c)* Local maxima were detected above a user-defined threshold using an implementation by E. Dufresne (2). *(d)* Brightness-weighted centroids of all candidates are calculated. Particle positions are kept depending on the spot size and the local SNR. The local SNR of a particle was defined as the ratio of its signal above local background to the standard deviation the local background. *(e)* Particle positions were subsequently linked to recover their trajectories using an algorithm based on the work of Crocker and Grier (1). *(f)* Typical trajectories obtained from LPS-bound receptors tracked over 100 frames, recorded at Δ*t* = 33 ms intervals.

**Supplementary Note S2** Testing of spot detection and localization procedures

We tested how our detection efficiency and our localization precision depend on the SNR using simulated data. We generated images of multiple isolated sub-resolution particles (Airy disc radius = 2 pixel), added noise, and compared the subsequent detection results obtained from the centroid calculation and the more elaborate Gaussian fitting with the original particle positions. The SNR of an image was defined as the average SNR of all particles present in the image, with the SNR of a particle being the ratio of its signal above local background to the standard deviation the local background.


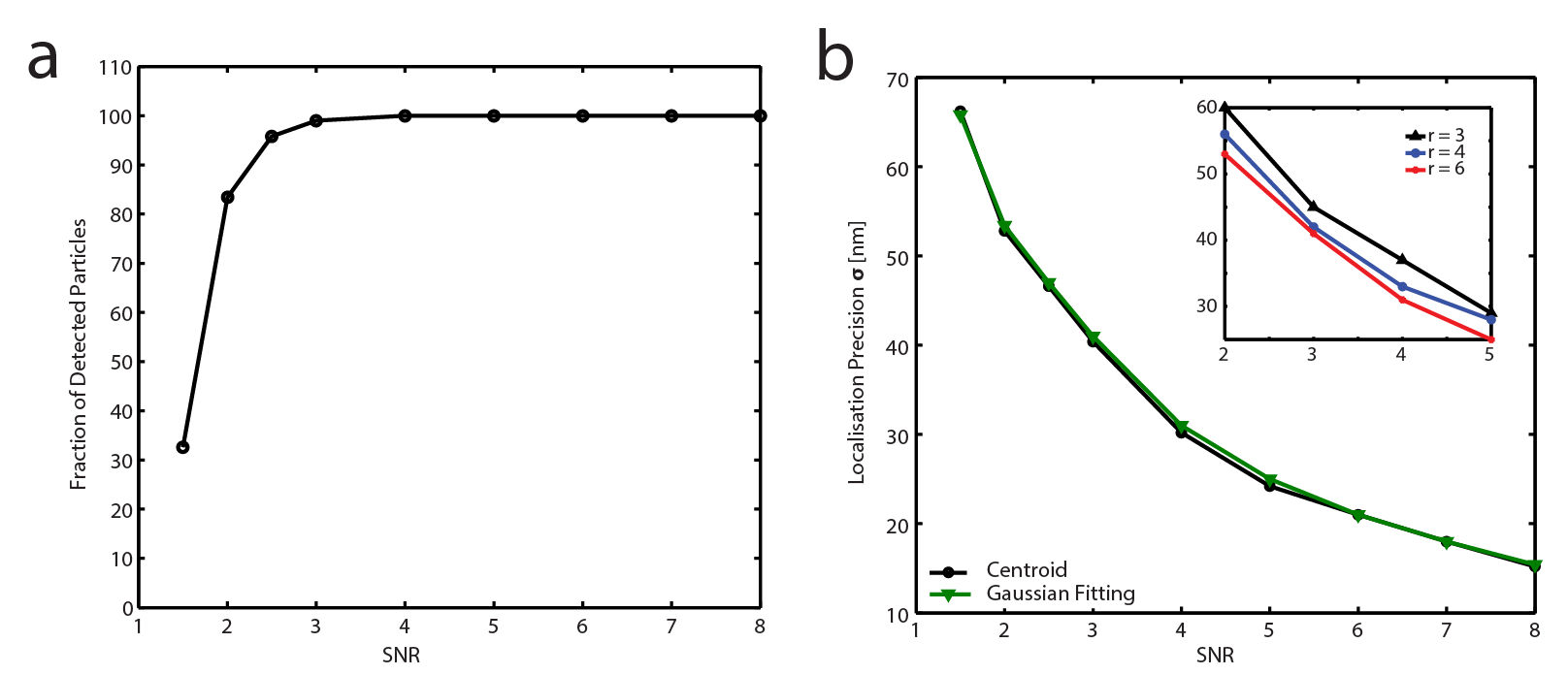


Figure S6 The detection efficiency (number of true positives / ground truth) and localization precision (distance of true positives to ground truth). *(a)* The fraction of detected particles as a function of the particles’ SNR for isolated particles. *(b)* The localization precision for centroid (black line) and Gaussian (green line) localization procedures as a function of the SNR for isolated sub-resolution particles and for particles located at various distances = *d* *x* *radius of Airy disk* from each other (insert). 15000 particles are simulated for each data point.

Fig. S6 *a* shows that 100% of the spots were detected for a SNR greater than 4. As expected, the detection efficiency was independent on the localization procedure (centroid vs Gaussian Fitting) and due to the perfect overlay only one curve is shown. In contrast, for SNR < 4 the localization precision is expected to be better using a fit of a Gaussian curve (3). We found, however, that localizing the spots using the more elaborate Gaussian approach did not increase the localization precision (Fig. S6 *b*). Both curves nearly overlap, and the difference in precision is marginal. Hence we decided to use the simpler and less computational expensive approach of centroid calculation for further analysis. As to be expected, the SNR inversely correlated with the localization precision. This correlation did not strongly depend on the mean particle-particle distance (insert). At a SNR of 11 (as typically seen in the data analyzed in this study) the theoretical localization precision is approximately 15 nm.

**Supplementary Note S3:** Comparison of MSD and JD Analysis using simulated data


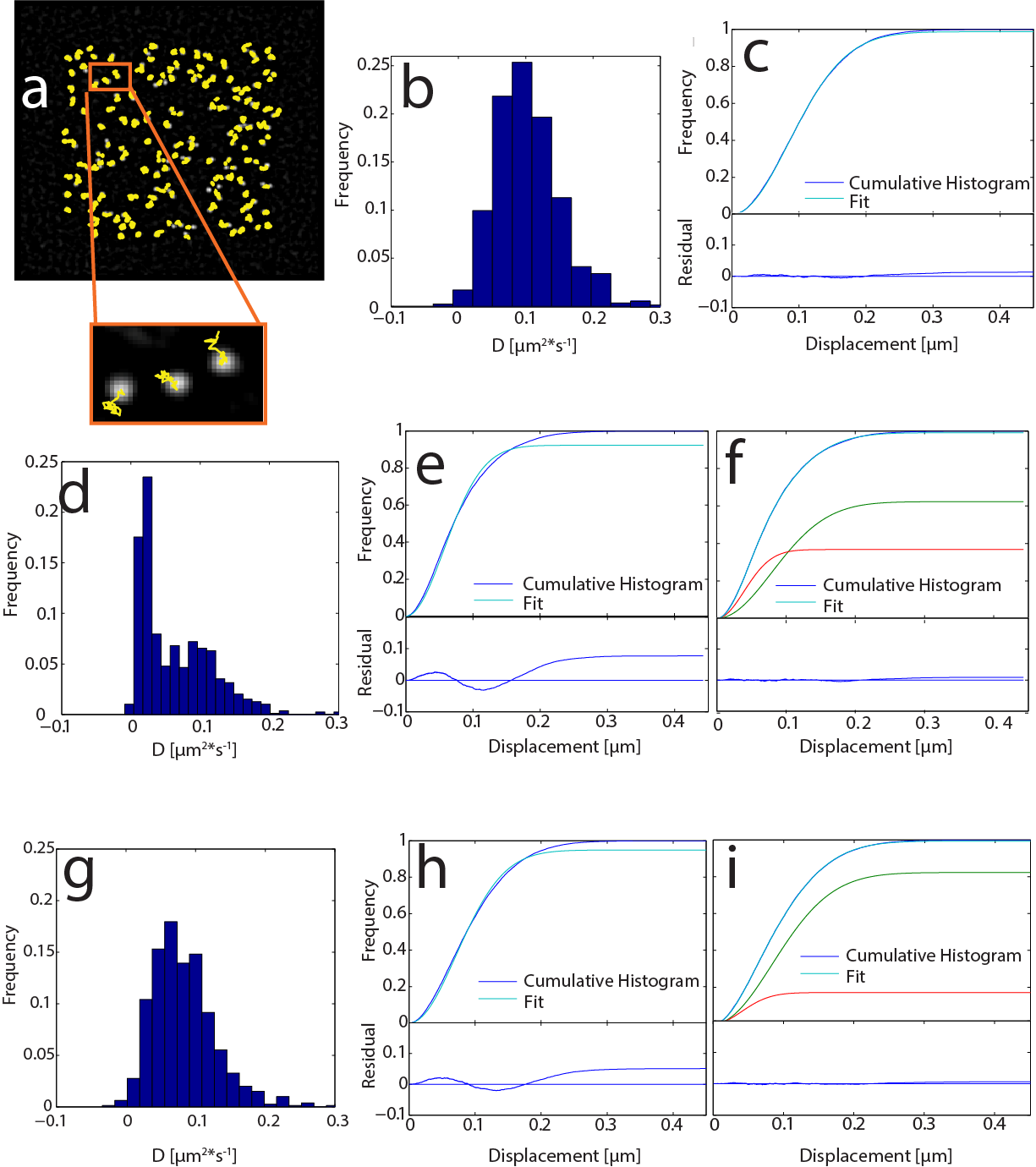


Figure S7 Comparison of MSD and JD Analysis for varying mobile and immobile populations. *(a,b,c)* All particles belong to the same mobile population. *(d,e,f)* 50 % of the particles are mobile, and 50 % immobile. *(g,h,i)* 50% of particles undergo a motion change (mobile → immobile). *(a)* Representative ensemble of simulated trajectories belonging to a single mobility population. *(b)* Representative histogram of diffusion coefficients obtained from MSD Analysis and *(c)* representative cumulative histogram for the corresponding Jump Distances (JD) for one population with a single, constant diffusion coefficient (*D_m_(ground truth, GT)* = 0.1 μm^2^/s). Best Fits according to equation 6 are shown. A single population fit extracts *D_m_(output)* = 0.11 μm^2^/s *(d)* MSD Histogram for two populations (*D_im_(GT)*= 0.02 μm^2^/s and *D_m_(GT)*= 0.1 μm^2^/s). *(e)* Single population fit: *D_m_(output)* = 0.05 μm^2^/s. *(f)* two population fit: *D_m_(output)* = 0.10 μm^2^/s, *D_im_(output)* = 0.02 μm^2^/s, *f_m_* = 0. 63; red and green lines show the two fit components *(g)* MSD Histogram for 50% of particles undergoing a motion change (*D_m_* 🡪 *D_im_*, or *D_m_* 🡪 *D_im_* 🡪 *D_m_*) at some point during the trajectory; *(h)* Single population fit: *D_m_(output)* = 0.08 μm^2^/s, *(i)* two population fit: *D_m_ (output)* = 0.11 μm^2^/s, *D_im_(output)* = 0.02 μm^2^/s; red and green lines show the two fit components. Representative distributions are based on analyzing 750 simulated trajectories, with SNR = 6, *σ* = 29 nm, *a* = 1060 nm, Δ*t* = 33 ms and *β* = 4.

We tested both trajectory analysis approaches using sets of 750 simulated trajectories (typical number obtained by SPT experiments). Trajectories (Fig. S7 a) can be analyzed by calculating individual diffusion coefficients for each trajectory (Fig. S7 *b*). The mean over all diffusion coefficients *<D>* describes the ensemble. The JD approach fits the obtained jump distance distribution with the respective theoretical expression (Equation 6), and dissects different mobility populations (Fig. S7 *c*). It is noteworthy that in case of two mobility fractions the histogram of individual *D*-values (Fig. S7 *d*) should show two peaks, however more trajectories than 750 would be needed to distinguish the distributions reliably. The JD approach, however, performed well even for a data set of this size. Whereas fitting the data with one mobility population showed systematic deviations in the residual (Fig. S7 *e*), fitting with two mobility populations gave no systematic deviations (Fig. S7 *f*) and recovered *D_m_* reliably (*D_m_(GT)* = *D_m_(output)* = 0.1 μm^2^/s). If a population with a motion change was present, the MSD approach failed independent of the size of the data set, as two populations could not be seen in the respective histogram (Fig. S7 *g*). The JD approach was still able to reliably dissect both fractions (Fig. S7 *i*), again a comparison of the single- and the two population fit (Fig. S7 h,*i*) showed that a fit of two mobility populations had to be used.

We further note that in comparison to the MSD method which requires complete trajectories, the JD approach only requires correct connections of successive frames. Hence this approach is less sensitive on the tracking function’s ability to recover complete trajectories.

**Supplementary Note S4** Measuring the localization precision

Lipopolysaccharide (LPS) labeled with AlexaFluor®488 was diluted in PBS and imaged on a Piranha cleaned glass coverslip. Interaction of LPS with glass immobilized the construct and no further fixation was required. For each particle, its center position over time was determined using the spot detection and tracking algorithm described. We define the standard deviation of the temporal center positions as the particle’s localization precision *σ*. Only trajectories with a minimum length of 100 frames were analyzed.


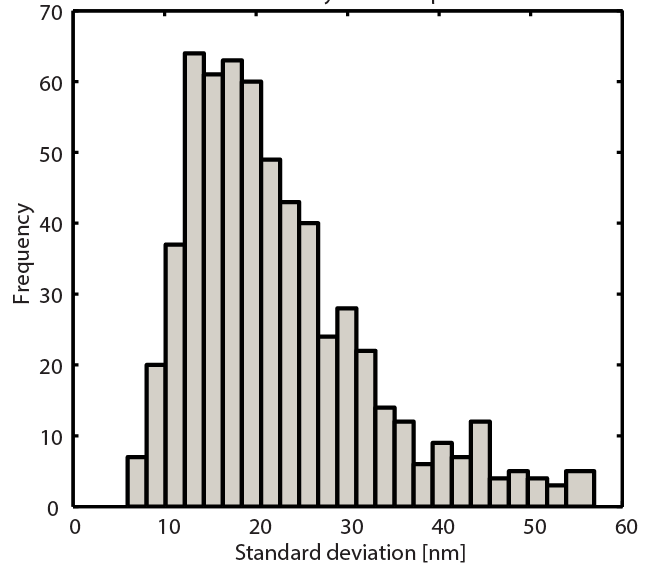


Figure S8 Localization precision of the imaging system. Histogram of the localization precision σ of individual immobilized LPS labeled with AlexaFluor®488. 599 particles with an SNR of 14 were analyzed. The mean value was found to be *<σ>* = 22 ± 10 nm.

Analyzing 599 particles with an SNR of 14, we obtained *<σ>* = 22 ± 10 nm. With *σ^2^ = 4Dt*, this corresponds to *D_im_* = 0.004 ± 0.003 μm^2^/s. Since the SNR of immobilized LPS was slightly higher than for receptor data (SNR = 11), a better estimate of the localization precision is *σ* = 25 ± 10 nm (*D_im_* = 0.005 ± 0.004). In addition, real receptor data is not expected to be perfectly immobilized as the labeled molecule is still part of a larger-scale dynamic system: the cell membrane fluctuates, and receptors might appear to be immobile because they are trapped in small membrane compartments, yet this means they still move within the compartment. We thus estimated the apparent *D_im_* to be in the range of *D_im_* = 0.02 μm^2^/s.

**Supplementary Note S5** Test of biological activity of AlexaFluor®488 labeled LPS

Toll-like receptor 4 (TLR4), MD-2 and CD14 are all required for efficient recognition and signaling to LPS. HEK293 cells do not express these receptors endogenously, and so provide a convenient and highly controlled system for detailed LPS pharmacology analysis when transfected with these receptors. HEK293 cells were plated on a 96-well plate and transiently transfected with human TLR4, MD-2 and CD14 together with two reporter plasmids: NF-B-luc, a firefly luciferase under an NF-B promoter, and phRG-TK, a constitutively expressed Renilla luciferase that normalizes for cell death, cell number and transfection efficiency. Cells were then stimulated 48 hours later in the presence of 0.1% FCS for six hours with labeled E. coli LPS, unlabeled E. coli LPS, or medium alone, and then lysed using Passive Lysis Buffer (Promega). Luminescence of the cell lysate following addition of the respective substrates (luciferin and coelenterazine) was measured by a luminometer, and NF-B activity inferred for each well by normalization of the firefly luciferase reading to the Renilla luciferase reading for that well (relative increase in luciferase = firefly luciferase luminescence/Rennila luciferase luminescence). NF-κB activation as measured by luciferase activity for labeled LPS was comparable to unlabeled LPS, although the concentration dependency was less pronounced for labeled LPS.

**
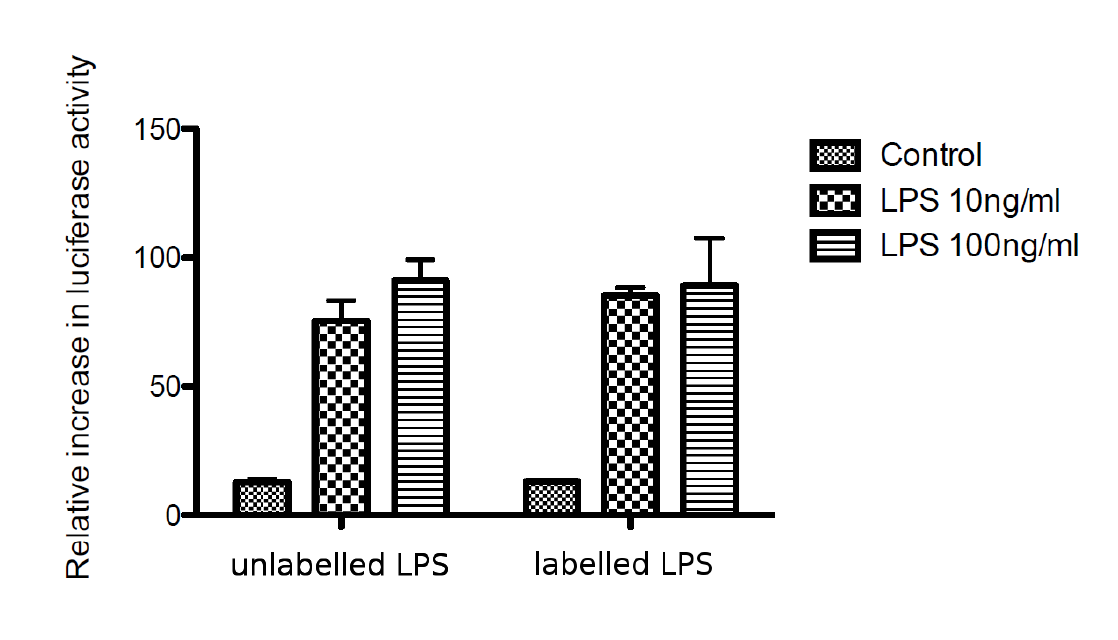
**Figure S9. LPS induced activation of the TLR4 pathway as indicated by an increase in luciferase activity is comparable for unlabeled and labeled (Alexa®488) LPS. This result is consistent over the two LPS concentrations tested (10 and 100 ng/ml). Data are from a single representative experiment (n=3 experiments), and are expressed as the mean of triplicate wells ± triplicate SE.

**Supplementary Movies**

**Movie S1** Representative Movie showing simulated receptor motion (raw data). All particles belong to the same mobility population (*D_m_* = 0.1 μm^2^/s). The duration of the movie in real-time is 1s and it is played back at 4x (7 frames per second).

**Movie S2** Same video as Movie S1 after application of a band-pass filter. Linked particles are marked with a number throughout the video. The ensemble plot of the trajectories is shown below:


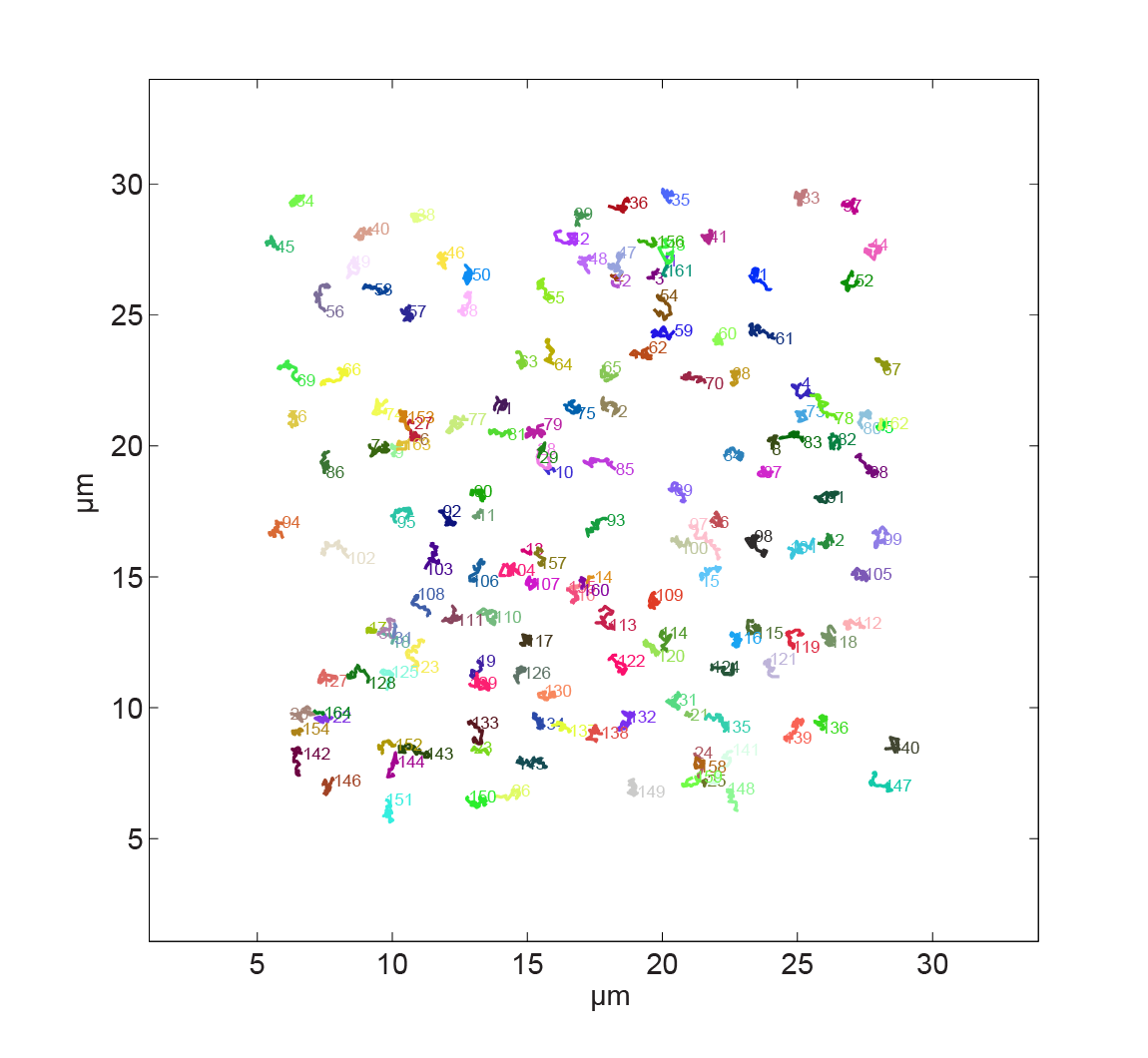


**Movie S3** Movie typically obtained for AlexaFluor®488 labelled LPS on wild-type macrophages (raw data). The duration of the movie in real-time is 4.2s and it is played back at 4x (7 frames per second).

**Movie S4** Same video as Movie S3 after application of a band-pass filter. Linked particles are marked with a number throughout the video. The ensemble plot of the trajectories is shown below:


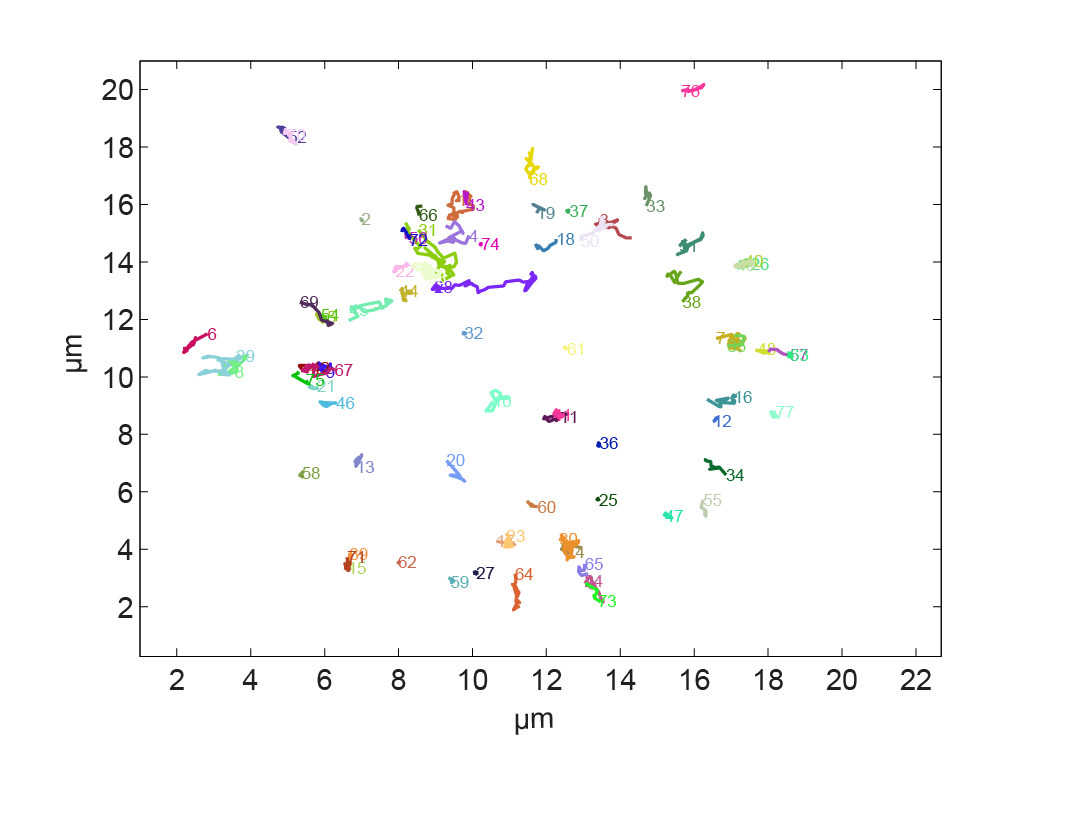


**References**

1. Croker, J.C., and D.G. Grier. 1996. Methods of Digital Video Microscopy for Colloidal Studies. J Colloid Interface Sci. 310: 298–310.

2. The Matlab Particle Tracking Code Repository website, Available: http://physics.georgetown.edu/matlab/. Accessed 2013 Apr 16.

3. Cheezum, M.K., W.F. Walker, and W.H. Guilford. 2001. Quantitative comparison of algorithms for tracking single fluorescent particles. Biophys J. 81: 2378–88.
